# Floating Matrix Tablets of Domperidone Formulation and Optimization Using Simplex Lattice Design

**Published:** 2011

**Authors:** Shailesh Prajapati, Laxmanbhai Patel, Chhaganbhai Patel

**Affiliations:** a *Department of Pharmaceutics, Shri Sarvajanik Pharmacy College, Mehsana, Gujarat, India.*; b *Department of Pharmaceutics, C. U. Shah College Institute of Pharmacy and Research Wadhwan, Gujarat, India.*

**Keywords:** Domperidone, Floating matrix tablets, Simplex lattice design, Release kinetics, Polyethylene oxide, Hydroxypropyl methylcellulose, Floating lag time, Total floating time.

## Abstract

The purpose of this research was to prepare a floating matrix tablet containing domperidone as a model drug. Polyethylene oxide (PEO) and hydroxypropyl methylcellulose (HPMC) were evaluated for matrix-forming properties. A simplex lattice design was applied to systemically optimize the drug release profile. The amounts of PEO WSR 303, HPMC K15M and sodium bicarbonate were selected as independent variables and floating lag time, time required to release 50% of drug (t_50_) and 80% of drug (t_80_), diffusion coefficient (n) and release rate (k) as dependent variables. The amount of PEO and HPMC both had significant influence on the dependent variables. It was found that the content of PEO had dominating role as drug release controlling factor, but using suitable concentration of sodium bicarbonate, one can tailor the desired drug release from hydrophilic matrixes. The linear regression analysis and model fitting showed that all these formulations followed Korsmeyer and Peppas model, which had a higher value of correlation coefficient (r). The tablets of promising formulation were found to be stable for 3 months under accelerated (40°C / 75% RH) stability testing.

## Introduction

Rapid gastrointestinal transit could result in incomplete drug release from the device above the absorption zone leading to diminished efficacy of the administered dose (1). Therefore, different approaches have been proposed to retain the dosage form in the stomach. These include bioadhesive systems, (2) swelling and expanding systems, (3, 4) and floating systems (5, 6). Large single-unit dosage forms undergo significant swelling after oral administration and the swollen matrix inhibits the gastric emptying even at an uncontractile state of the pyloric sphincter. Park and Park reported medicated polymeric sheets and swelling of balloon hydrogels (7). But the swelling and expanding systems may show the hazard of permanent retention. Bioadhesive systems may cause problems such as irritation of the mucous layer owing to high localized concentration of the drug (8). Hydrodynamically balanced systems were designed using effervescent mixtures.

In recent years, polyethylene oxide (PEO) has attracted much attention as a polymeric excipient that can be used in formulations for different purposes. For instance, formulations with PEO have been extruded to make different products such as swellable and erodible implants (9), scaffolds for tissue engineering (10), or, to be used in the production of micelles with amphiphilic drugs, when solid dispersions incorporating these drugs are placed in aqueous environments (11). However, PEOs are mostly used to produce controlled release solid dosage forms such as matrixes, reservoirs, or coated cores (12, 13, 14). Due to their chemical structure, PEOs are among various hydrophilic polymers that, in the presence of water, control the release of the active moiety either by swelling (large molecular weight; > 2 MDa (mega Dalton)) or by eroding and swelling (small molecular weight; < 0.9 MDa), forming a hydrogel. In both cases, water triggers the process starting the erosion and/or the swelling processes. All this attention to PEOs is due to the consequence of their physical and chemical stability, compressibility, high swelling ability, and good solubility in water. Thus, PEOs have been proposed as alternatives to cellulose or other ethylene glycol derivatives in the production of tablets or granules.

Domperidone is a synthetic benzimidazole compound that acts as a dopamine D2 receptor antagonist. Its localization outside the blood-brain barrier and antiemetic properties has made it a useful adjunct in therapy for Parkinson’s disease. There has been renewed interest in antidopaminergic prokinetic agents since the withdrawal of cisapride, a 5-HT4 agonist, from the market. Domperidone is also used as a prokinetic agent for treatment of upper gastrointestinal motility disorders (15, 16). It continues to be an attractive alternative to metoclopramide because of its fewer neurological side effects. Patients receiving domperidone or other prokinetic agents for diabetic gastropathy or gastroparesis should also be managing diet, lifestyle, and other medications to optimize gastric motility (17). It is rapidly absorbed from the stomach and the upper part of the gastrointestinal tract (18) after the oral administration and few side effects have been reported (15, 16). It is a weak base with good solubility in acidic pH but in alkaline pH solubility is significantly reduced. Oral controlled release dosage forms containing drug, which is a weak base, are exposed to environments of increasing pH and the poorly-soluble freebase may be precipitated within the formulation in the intestinal fluid. Precipitated drug is no longer capable of being released from formulation (19, 20). The short biological half-life of drug (7 h) also favors development of a sustained release formulation. 

The major objective of the present investigation was to develop a gastroretentive drug delivery system containing domperidone using simplex lattice design as an optimization technique.

## Experimental

*Materials*

Domperidone was a kind gift from Maan Pharmaceutical Ltd (Mehsana, India). Polyethylene oxide WSR 303 (Polyox^®^ WSR 303, mw = 7×10^6^) was received as a gift sample from Dow Chemical company, New Jersey (USA), Hydroxypropyl methylcellulose (HPMC K15 M), and sodium bicarbonate were procured from Laser Chemicals (Ahmedabad, India). Magnesium stearate and talc were purchased from Apex Chemicals (Ahmedabad, India). All other ingredients used were of analytical grade and were used as received.

*Methods*

*Preparation of domperidone floating tablets*

Domperidone, the required quantity of polymers (Polyox^®^ WSR 303 and HPMC K15M), sodium bicarbonate and starch were mixed in mortar by spatula for 15 min. The powder blend was then lubricated with talc and magnesium stearate and compressed in tablets using 8 mm flat-face round tooling on rotary tablet press (Rimek, India, Ahmedabad). Compression force was adjusted to obtain tablets with hardness in range of 4-5 Kg/cm^2^. The tablets weighed 145 ± 2 mg, had a round flat-face with average diameter 8 ± 0.1 mm and a thickness of 2.5 ± 0.2 mm.

*Simplex lattice design*

A simplex lattice design (21) was adopted to optimize the formulation variables. In this design, three factors were evaluated by changing their concentrations simultaneously and keeping their total concentration constant.

**Figure 1:**
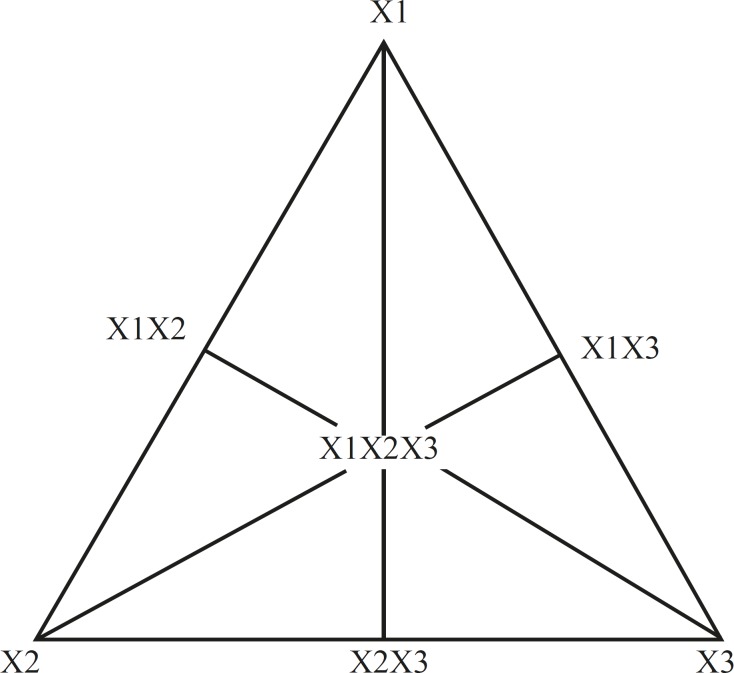
Equilateral triangle representing simplex lattice design for 3 components

The simplex lattice design for a 3-component system is represented by an equilateral triangle in 2-dimensional space (Figure 1). Seven batches (S_1_-S_7_) were prepared (Table 1) by taking three independent variables; one at each vertex (X_1_, X_2_, X_3_), one at the halfway point between vertices (X_1_X_2_, X_2_X_3_, X_1_X_3_), and one at the center point (X_1_X_2_X_3_). Each vertex represents a formulation containing the maximum amount of 1 component, with the other 2 components at a minimum level. The halfway point between the 2 vertices represents a formulation containing the average of the minimum and maximum amounts of the 2 ingredients represented by 2 vertices. The center point represents a formulation containing one third of each ingredient.

**Table 1:** Formulation and evaluation of batches in simplex lattice design

	**Transformed fraction of variables **			**Dependent Variables **								
**Batch Code **	**X**_1_	**X**_2 _	**X**_3 _	**FLT ± SD ** **(sec) **		**t**_50_% **± SD ** **(h) **		**t**_80_% **± SD ** **(h) **		**n ± SD **		**k ± SD **
**S**_1_	1	0	0	20 ± 2		9.583 ± 1.9		12.344 ± 2.2		0.733 ± 0.045		6.445 ± 0.3
**S**_2_	0	1	0	55 ± 3		12.684 ± 2.3		17.435 ± 2.6		0.591 ± 0.007		9.853 ± 1.2
**S**_3_	0	0	1	10 ± 4		11.702 ± 0.8		21.527 ± 0.8		0.620 ± 0.004		9.929 ± 0.4
**S**_4 _	0.5	0.5	0	35 ± 5		17.077 ± 1.7		26.350 ± 1.7		0.513 ± 0.032		14.435 ± 2.1
**S**_5 _	0	0.5	0.5	98 ± 3		18.11 ± 1.4		28.49 ± 1.1		0.489 ± 0.0019		15.402 ± 0.3
**S**_6_	0.5	0	0.5	25 ± 2		11.194 ± 0.5		23.811 ± 0.7		0.635 ± 0.0021		10.386 ± 0.7
**S**_7 _	0.33	0.33	0.33	39 ± 3		15.277 ± 1.2		23.071 ± 2.0		0.5748 ± 0.002		12.319 ± 1.8
					**Actual Value **							
					**Coded Value **		**X**_1_		**X**_2 _		**X**_3 _	
					1		60		30		20	
					0		50		20		10	

FLT: Floating lag time; SD: Standard deviation; t_50%_ and t_80%_: Time required for 50% and 80% drug dissolution; n: Diffusion coefficient; k: Release rate constant; X_1_: Amount of Polyethylene oxide WSR 303 (mg); X_2_: Amount of HPMC K15M (mg); X_3_: Amount of Sodium bicarbonate (mg). All batches contained 30 mg of domperidone, 20 mg of maize starch, 2% wt/wt of talc, and 1% wt/wt of magnesium stearate. Average weight of each tablet was 145 mg.

The amounts of matrixing agent (Polyethylene oxide WSR 303, X_1_), gelling agent, (HPMC K15M, X_2_), and gas-generating agent (sodium bicarbonate, X_3_) were selected as independent variables. Floating lag time (FLT), time required for 50% and 80% drug release (t_50_ and t_80 _respectively), Diffusion exponent (n), and release rate constant (k) were selected as dependent variables. 

A statistical model incorporating 7 interactive terms was used to evaluate the responses. 

Y = b_0_ + b_1_X_1_ + b_2_X_2 _+ b_3_X_3_ + b_1,2_X_1_X_2_+ b_2,3_X_2_X_3_ +b_1,3_X_1_X_3_ + b_1,2,3_X_1_X_2_X_3 _

Where Y is the dependent variable, b_0_ is the arithmetic mean response of the 7 runs, and bi is the estimated coefficient for the factor Xi. The main effects (X_1_, X_2_, and X_3_) represent the average result of changing 1 factor at a time from its low to high value. The interaction terms (X_1_X_2_, X_2_X_3_, X_1_X_3_, and X_1_X_2_X_3_) show how the response changes when 2 or more factors are simultaneously changed. The statistical analysis of the simplex lattice design batches was performed by multiple linear regression analysis using Microsoft Excel.

**Figure 2:**
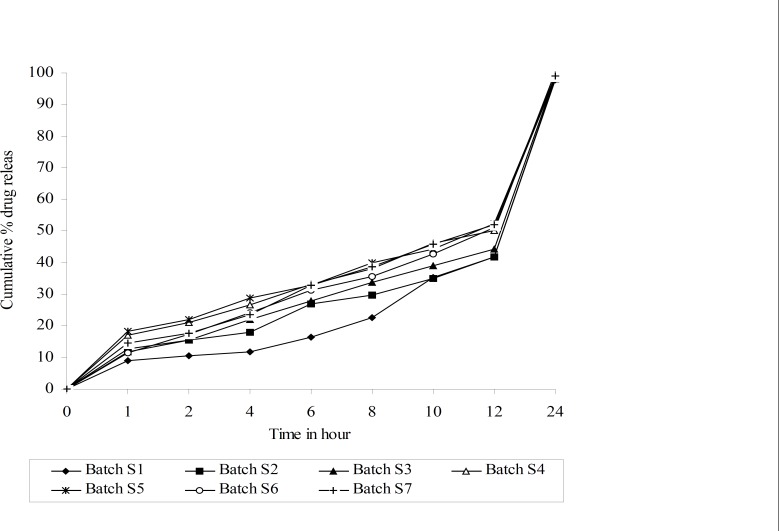
Drug release profiles of simplex lattice design batches

*In-vitro buoyancy studies*

The *in-vitro *buoyancy was determined by floating lag time as per the method described by Rosa *et al. *(22). The tablets were placed in a 100 mL glass beaker containing simulated 0.1N Hydrochloric acid, as per USP. The time required for the tablet to rise to the surface and float, was determined as the floating lag time.

*In-vitro dissolution studies*

The *in-vitro *dissolution study of domperidone tablets was performed using USP apparatus (model TDT-06T, Electrolab, Mumbai, India) fitted with paddles (50 rpm) at 37°C ± 0.5°C using Hydrochloric acid (pH 1.2, 900 mL) as a dissolution medium. At the predetermined time interval, 5 mL samples were withdrawn, filtered through a 0.45 m

*Calculation of immediate release part*

The pharmacokinetic parameters of domperidone were used to calculate a theoretical drug release profile for a 24 h dosage form. The immediate release part for sustained release domperidone was calculated using Equation 1 and was found to be 4.211 mg.

Immediate release part = (Css × Vd) / F (1)

Where, *C*_SS_ is steady-state plasma concentration (Average C_max_), V_d_ is volume of distribution, and *F *is fraction bioavailable. Hence, the formulation should release 4.211 mg (14.04%) of drug in 1 h like conventional tablets and 1.121 mg (3.74%) per hour up to 24 h. The similarity factor, f2, given by Scale Up and Pose Approval Changes (SUPAC) guidelines for modified release dosage form was used as a basis to compare dissolution profiles (24).

**Table 2:** Analysis of variance table for dependent variables from simplex lattice design

**Source**	**SS**	**DF**	**MS**	**F value**	**Prob**
***Floating lag time (FLT)***					
**Model**	4	5179.885	1294.971	28.29187	0.03443
**Residual**	2	91.54371	45.77185		
**Total**	6	5271.429			
**Time required for 50% drug release (t**_50%_ **)**					
**Model**	3	6.45989986	2.15329995	9.538526	0.048185
**Residual**	3	0.67724299	0.22574766		
**Total**	6	7.13714286			
**Time required for 80% drug release (t**_80%_ **)**					
**Model**	1	16.814736	16.814736	6.888313	0.046839
**Residual**	5	12.205264	2.4410528		
**Total**	6	29.02			

**Model**	3	0.03359082	0.01119694	35.0759	0.007769
**Residual**	3	0.00095766	0.00031922		
**Total**	6	0.03454848			
**Release rate constant (k)**					
**Model**	2	57.0932443	28.5466222	10.72798	0.024691
**Residual**	4	10.6437974	2.66094936		
**Total**	6	67.7370417			

DF: Degree of freedom; SS: Sum of square; MS: Mean of square; F: Fischer’s ratio

## Results and Discussion

Polyethylene oxide WSR 303 was selected as a matrixing agent to impart sufficient integrity of the tablets. HPMC K 15 M was selected as a gelling agent, considering its widespread applicability and excellent gelling activity in sustained release formulations. Sodium bicarbonate generates CO_2_ gas in the presence of hydrochloric acid, present in dissolution medium. The generated gas is trapped and protected within the gel (formed by hydration of HPMC), leading to decrease in density of the tablet. As the density of the tablet falls below 1 (density of water), the tablet becomes buoyant. It was observed that the increase in amount of Polyethylene oxide WSR 303, leads to decrease the cumulative percentage of drug release. Hence, it was decided to optimize the amount of polyethylene oxide WSR 303 between drug, polyethylene oxide WSR 303 1 : 2 ratio. As the amount of HPMC K15M was increased from drug to polymer (1 : 1 to 1 : 3 ratio), the floating lag time increased, indicating that a high amount of HPMC is undesirable to achieve low floating lag time. Below drug to polymer 1 : 1 ratio HPMC K 15M might not give sufficient strength to the matrix to prolong drug release up to 24 h. Hence, it was decided to optimize HPMC K 15 M for drug, HPMC K 15 M in 1 : 1 ratio. Twenty mg of sodium bicarbonate was optimized as CO_2 _producing agent from preliminary studies.

The values for Floating lag time (FLT), time required for 50% and 80% drug release (t_50% _and t_80%_ respectively), release rate constant (k) and diffusion component (n) for all 7 batches (S_1_-S_7_) showed a wide variation (Table 2). The data clearly indicate that the values of FLT, t_50%_, t_80%_, k and n are strongly dependent on the selected independent variables.

**Figure 3:**
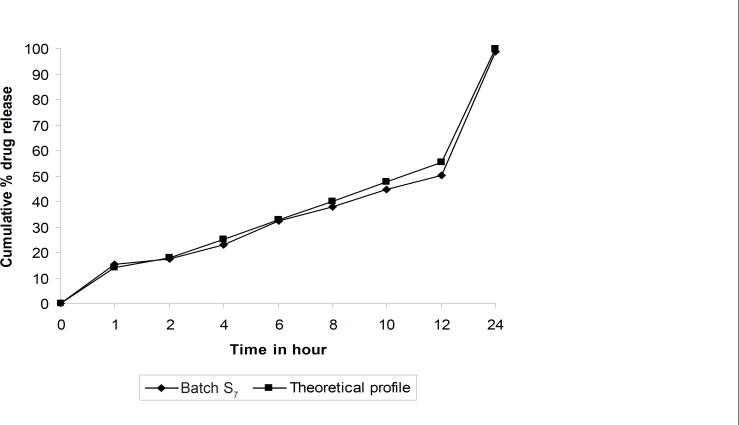
Comparison of *in-vitro *dissolution profiles of batch S_7_ and theoretical dissolution profile.

Dissolution profiles of all batches of factorial design were compared with theoretical dissolution profile. The results of similarity factor indicate that batches S^2^ to S^7^ fulfill the above criteria. But batch S_7_ showed highest f2 among all the batches. Hence, batch S_7 _more similar compare to other batches of simplex lattice design, similarity between theoretical dissolution profile and dissolution profile of S_7 _is shown in Figure 3.

The fitted equation relating the responses Floating lag time (FLT), time required for 50% and 80% drug release (t_50%_ and t_80%_ respectively), release rate constant (k) and diffusion component (n) to the transformed factor are shown in Equations 2, 3, 4, 5 and 6, respectively.

FLT = 98.7859 - 62.4770 × X_2_ - 87.4770 × X_3_ - 62.7759 × X_1_X_2_ - 132.7759 × X_2_X_3_

R - square = 0.98263 ( 2 ) 

t_50%_ = 12.4872 - 1.2714 × X_3_ - 9.6857 × X_1_X_2_ - 5.9428 × X_2_X_3_

R - square = 0.90511 ( 3 ) 

t_80%_ = 19.1078 + 17.2948 × X_1_X_2_

R - square = 0.9418883 ( 4 ) 

n = 0.6422 + 0.0676 × X_1_ - 0.6017 × X_1_X_2_ - 0.4456 × X_2_X_3_

R - square = 0.97228066 ( 5 ) 

k = 9.0676 + 22.9004 × X_1_X_2_ + 24.8700 × X_2_X_3_

R - square = 0.9180772 ( 6 ) 

The high value of correlation coefficient for FLT, t_50%_, t_80%_, n and k indicate good fit (Table 2). The polynomial equations can be used to draw the conclusions after considering the magnitude of coefficient and the mathematical sign that it carries (*i.e.*, positive or negative). 

**Figure 4:**
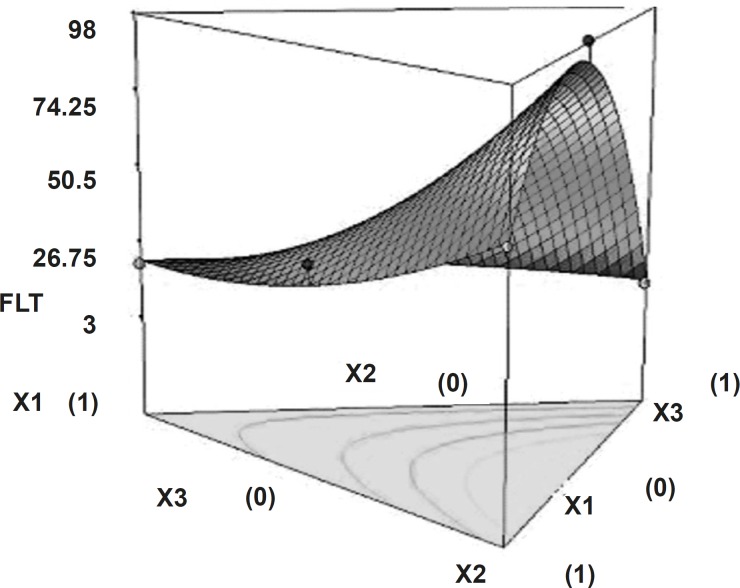
Response surface plot (3D) showing the effect of the amount of PEO, HPMC and sodium bicarbonate on floating lag time

Tablets of all batches (S_1_ to S_7_) had floating lag time varies from 10 sec to 98 sec. Polynomial equation for floating lag time (Equation 2) suggests that the amount of sodium bicarbonate and HPMC K15M has more significant effect on floating lag time. It may due to the interaction amongst gas generating agent (NaHCO_3_), dissolution medium (0.1 N HCl, pH of 1.2) reduce FLT, and hydrophilic nature of HPMC, which produce easy swelling of tablets. Figure 4 shows the 3D surface plot of the amount of PEO WSR 303 (X_1_), amount of HPMC K 15 M (X_2_) and amount of sodium bicarbonate (X_3_) versus FLT. The plot was drawn using State-Ease (Design-Expert® version 7, Stat-Ease, Inc., Minneapolis, MN 55413). The data demonstrate that X_1_, X_2_ and X_3_ affect the floating lag time. It may also be concluded that the low level of X_1_ (amount of PEO WSR 303) and the high level of X_3_ (amount of sodium bicarbonate) favor the low floating lag time. The high value of X_2_X_3 _coefficient also suggests that the interaction between X_2_ and X_3_ has a significant effect on FLT. It can be concluded that the FLT changed by appropriate selection of the X_2_ and X_3_ levels.

**Figure 5:**
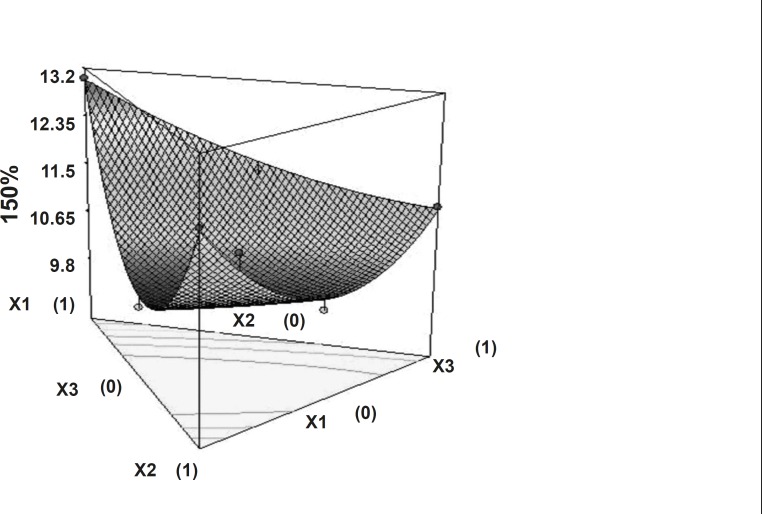
Response surface plot (3D) showing the effect of the amount of PEO, HPMC and sodium bicarbonate on t_50%_.

**Figure 6:**
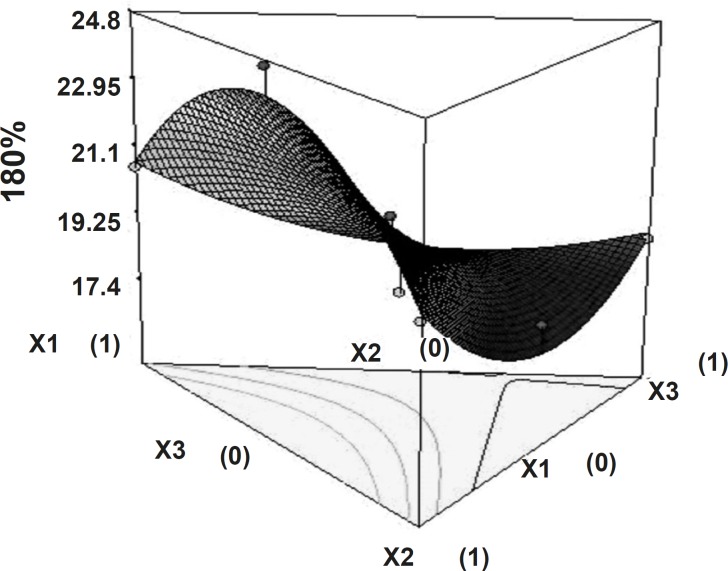
Response surface plot (3D) showing the effect of the amount of PEO, HPMC and sodium bicarbonate on t_80%_

The time required to release 50% of drug (t_50%_) and the time required to release 80% of drug (t_80%_) showed wide variation (Table 1). Figures 5 and 6 show the 3D surface plot of the amount of PEO WSR 303 (X_1_), HPMC K 15 M (X_2_) and sodium bicarbonate (X_3_) versus t50% and t80%, respectively. The data clearly indicate that the dependent variables (t_50%_, t_80%_) are strongly dependent on the independent variables. The fitted equation relating the response t_50%_ and t_80%_ to the transformed factors are shown in Equations 3 and 4. Data of t_50%_ and t_80%_ clearly indicate that increase in the amount of sodium bicarbonate leads to decrease in the time required to 50% drug release. It may due to pores formation in tablet by sodium bicarbonate which produce CO_2_ when interacts with dissolution medium. The high value of X_1_X_2_ coefficient also suggests that the interaction between X_1_ and X_2_ has a significant effect on t_80%_. It can be concluded that the t_80% _changed by an appropriate selection of the X_1_ and X_2_ levels.

**Figure 7:**
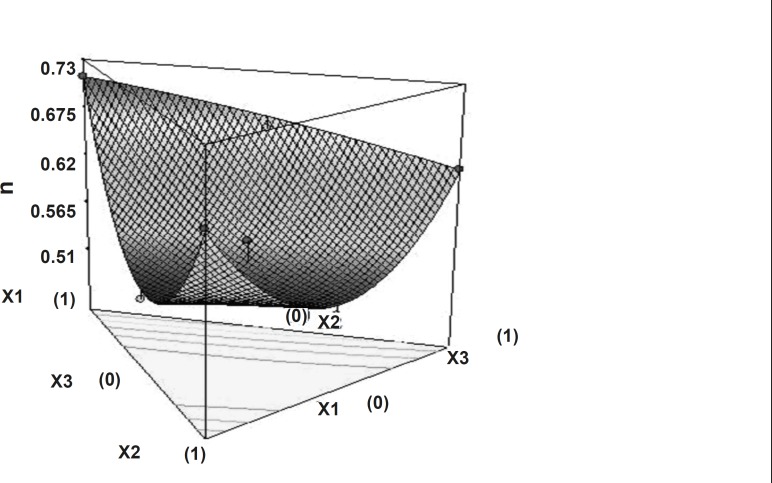
Response surface plot (3D) showing the effect of the amount of PEO, HPMC and sodium bicarbonate on diffusion exponent (n).

Dissolution profiles were fitted with the power law equation given by Korsmeyer and Peppas^24^. Diffusion exponent value varies from 0.489 to 0.7332 indicate that drug release pattern anomalous involves the combination of swelling, diffusion and/or erosion of matrixes. This might be due to the poor water solubility of domperidone as well as the difference exists in characteristics of polymers. Non-linear relationship was obtained between the diffusion exponent and the two independent variables. Figure 7 shows the 3D surface plot of the amount of PEO WSR 303 (X_1_), HPMC K 15 M (X_2_) and sodium bicarbonate (X_3_) versus diffusion exponent.

Release rate constant showed that independent factors had significant influence (p < 0.05).

**Figure 8:**
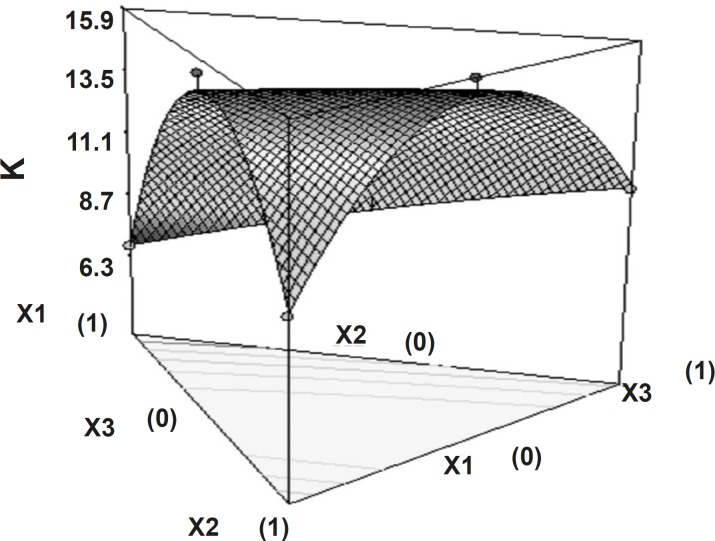
Response surface plot (3D) showing the effect of the amount of PEO, HPMC and sodium bicarbonate on release rate constant (k).

The high value of X_1_X_2_ and X_2_X_3_ coefficient also suggests that the interaction between X_1_X_2_ and X_2_X_3 _has a significant effect on release rate constant. It can be concluded that the release rate constant changed by appropriate selection of the X_1_, X_2_ and X_3_ levels. Figure 8 shows the 3D surface plot of the amount of PEO WSR 303 (X_1_), HPMC K 15 M (X_2_) and sodium bicarbonate (X_3_) versus release rate constant.

## Conclusion

The amount of PEO and HPMC both had significant influence on the dependent variables. It was concluded that the content of PEO had a dominating role as drug release controlling factor, but using suitable concentration of sodium bicarbonate, one can tailor the desired drug release from hydrophilic matrixes for the development of floating tablets.
